# A P4HA2 hypoxia signature derived from single cell atlas stratifies conserved subtypes with prognostic significance in cervical squamous cell carcinoma

**DOI:** 10.1186/s12885-026-15597-z

**Published:** 2026-01-21

**Authors:** Xiaojiao Li, Xinyuan Zhang, Yilin Dai, Fanwei Huang, Rui Wei, Xiaoyuan Huang, Ding Ma, Fei Li, Xi Li

**Affiliations:** 1 Department of Gynecological Oncology, Tongji Hospital, Tongji Medical College, Huazhong University of Science and Technology, Wuhan, China; 2 National Clinical Research Center for Obstetrics and Gynecology, Cancer Biology Research Center (Key Laboratory of the Ministry of Education), Tongji Hospital, Tongji Medical College, Huazhong University of Science and Technology, Wuhan, China

**Keywords:** Cervical squamous cell carcinoma, Hypoxia, Prognosis, scRNA-seq, Bulk RNA-seq, HIF-1α, Machine learning

## Abstract

**Background:**

Cervical cancer, especially cervical squamous cell carcinoma (CSCC), is a major public health issue in low- and middle-income countries, with advanced recurrence and metastasis linked to poor prognosis. It shows significant intra-tumoral phenotypic heterogeneity and plasticity.

**Methods:**

We analyzed the single-cell RNA sequencing data of cervical squamous cell carcinoma available in the Gene Expression Database (GEO) and identified the tumor cell subtype exhibiting hypoxic characteristics. We extracted differentially expressed genes (HRDEGs) between this hypoxia-related cluster and other tumor cells. Based on the CSCC bulk RNA sequencing data from the Cancer Genome Atlas (TCGA), this subtype was confirmed to be closely associated with poor prognosis in CSCC.101 combinations consisting of 10 machine learning models were used for screening prognostic biomarkers in HRDEGs, and a hypoxia signature was established by multivariate COX regression.

**Results:**

The hypoxia signature, validated using external GEO datasets, was significantly correlated with tumor invasiveness. Further analysis demonstrated that immune infiltration and responses to both chemotherapy and immunotherapy are associated with the hypoxia signature. In addition, the key gene P4HA2 in the hypoxia signature has been demonstrated to be involved in the regulation of malignant phenotypes of tumor cells and the regulation of HIF-1α stability.

**Conclusions:**

Overall, this hypoxia signature is a promising independent prognostic factor, provides potential biomarkers for the prognosis of CSCC and may guide future investigations into patient stratification.

**Supplementary Information:**

The online version contains supplementary material available at 10.1186/s12885-026-15597-z.

## Introduction

Cervical cancer (CC) was the fourth most common cancer in women worldwide [1, 2]. Although the system implementation of HPV vaccines has reduced the incidence and mortality of cancer substantially, the prognosis for patients with advanced-stage, recurrent, and metastatic CC remains poor [3, 4]. An estimation of 37% CC patients were diagnosed with locally advanced disease, standardly treated with concurrent chemoradiotherapy (CCRT) [5], among which, around 30% of post-treatment patients would develop disease recurrence or distant metastases [6]. Additionally, more than 10% CC cases were comprised of stage IVB disease posing an even more challenging treatment scenario [7–11]. In this context, neoadjuvant chemotherapy (NACT) followed by radical hysterectomy has been proposed as an alternative to CCRT, its major drawback is the potential delay of definitive local treatment in patients who are resistant to chemotherapy [6]. To circumvent this constraint in current clinical practice, early risk stratification and timely intervention are increasingly recognized as essential for delivering precise and individualized treatment. A promising strategy to achieve this is the identification and validation of novel biomarkers with prognostic and predictive value, which may reveal targetable molecular pathways and facilitate personalized cancer therapy [12].

Cervical squamous cell carcinoma (CSCC), accounting for 80% of CC, exhibits significant intra-tumor heterogeneity encompassing morphology, transcriptomic profiles, epigenomic modifications, metabolic activity, and accordingly treatment responses [13, 14]. This variability complicates current treatment approaches, as it limits their ability and increases the likelihood of treatment resistance, thereby underscoring the importance of biomarker-driven strategies for precision therapy.

Within the heterogeneous solid tumor microenvironment, the hypoxic propensity of CSCC is widely revealed to be highly involved in therapeutic responses across multiple modalities [15–17]. In contrast to normal uterine cervix tissue, consuming oxygen, CSCC is depicted with a hypoxia-glycolysis-acidosis paradigm due to the perturbation and deficiency in microcirculation [18]. This in turn generates tumor heterogeneity by causing uneven oxygen distribution [19]. This oxygen deprivation acts indeed as a surviving stressor compelling cancer cells to adapt to this unfavorable energy condition by prompting a variety of genes as *TP53*, *HIF1A* or *GLUT1* to evade apoptosis or necrosis [20]. Along with this adaptation, cancer cells re-allocate their energy turning out to mediate various essential processes such as proliferation, and angiogenesis, promoting tumor invasion, metastasis and treatment resistance in terms of chemotherapy, radiotherapy, and immunotherapy [21–23]. Therefore, efforts are extensively underway to decipher the role of hypoxia in CSCC to understand the tumor evolution and immune activity modulation, and consequently, improve the patient outcomes.

Building on these efforts, high-throughput sequencing allows single-cell resolution studies to elucidate hypoxia-driven cancer cell heterogeneity. Guo C. et al. presented the hypoxia role in modulating macrophage polarization in 2 CCSC samples [24]. Qiu J. et al. unveiled a hypoxia related clusters as a progenitor of CC by preforming a comprehensive single-cell atlas of a total of 17 samples including 3 CCSC cases [25]. Although these studies have proposed elaborate molecular mechanisms supporting tumor survival and carcinogenesis, the case sizes enrolled in these studies were usually limited by the absence of follow-ups. Integration of single-cell sequencing and bulk RNA sequencing could constitute a good investigation strategy to broaden single-cell findings to larger populations. By applying 10 machine learning algorithms into 101 combinations, we visualized a significant relationship between a P4HA2-dominated hypoxia signature and poor outcomes of 297 CSCC patients in silico. In this respect, we further conducted Transwell migration/invasion assays and CCK-8 assays to evaluate the role of P4HA2 in cancer cell proliferation, infiltration and aggressiveness in three cervical cell lines.

Collectively, herein, we identify a prognostic relevant hypoxia signature in CSCC and provide mechanistic evidence supporting the potential of targeting hypoxia and P4HA2 as a therapeutic strategy, revealing a direction for future exploration of hypoxia-targeted therapy.

## Materials and methods

### Data acquisition

The scRNA-seq data GSE208653 and GSE197461 are publicly available datasets from a comprehensive gene expression database(GEO, https://www.ncbi.nlm.nih.gov/geo/). We removed samples of cervical adenocarcinoma, CIN, and normal cervical epithelium, and only retained 5 CSCC patient samples. We obtained RNA-seq data and clinical information of TCGA-CSCC (*n* = 246) and GSE52903 (*n* = 51), from the TCGA database and GEO dataset, respectively. In addition, datasets GSE100080, GSE7410, GSE26511, and GSE146114 were obtained from GEO to explore the correlation between risk scores and clinical staging.

### ScRNAseq datasets and processing

Single-cell RNA sequencing (scRNA-seq) data analysis was performed using the Python package Scanpy (https://scanpy.readthedocs.io/) [26]. The analysis commenced with a raw gene expression count matrix. Initial quality control involved filtering out cells expressing fewer than 300 or more than 1000 genes, as well as cells with mitochondrial gene expression greater than 25%. The remaining raw counts were normalized per cell to a total count of 10,000, log1p-transformed, and scaled to unit variance. We performed principal component analysis with 50 components. Meanwhile, the “bbknn” package(https://github.com/Teichlab/bbknn) was used to remove the batch effect of scRNA-seq raw data arising from technical variations (e.g. sequencing and cohort batches) [27]. A neighborhood graph was then computed based on the batch-corrected PCA embeddings, and unsupervised clustering was performed with a resolution parameter of 0.8, yielding 24 distinct cell clusters. These clusters were visualized in two dimensions using UMAP with parameters n_neighbors = 15 and min_dist = 0.5. Finally, cell cluster identity was annotated manually based on the expression patterns of well-established marker genes curated from the existing literature and public databases.

### Malignant cell identification

The “inferCNVpy” package (https://github.com/icbi-lab/infercnvpy) was utilized in Python to infer the copy number variation (CNV) of epithelial cells in integrated single-cell RNA sequencing (scRNA-seq) data. The analysis was performed by running the cnv.tl.infercnv function, using all T cells as the reference. Subsequently, a quantitative CNV score for each cell was computed with the cnv.tl.cnv_score function. To visualize the distribution of CNV scores across the cell population, the scores were visualized on a UMAP dimensionality reduction map. Finally, cell clusters with significantly high CNV scores were annotated as malignant epithelial cells on the UMAP. This approach effectively distinguished normal epithelial populations from malignant epithelial populations for downstream analyses.

### Expression programs of intratumoral heterogeneity

To evaluate tumor cells’ heterogeneity, we performed Non-negative matrix factorization (NMF) with the ”geneNMF” R package(https://github.com/carmonalab/GeneNMF) and determined the robust program with the k value set between 3:9, and determined the consensus programs in metaprograms (MPs) that can be stably identified. In addition, we select the top 30 genes of each MP as the feature genes, and use the scanpy.tl.score_genes function to calculate the MP scores of each cell, and assign each cancer cell to the MP cluster with the highest score and project it onto the UMAP map.

### DEGs and biological function and pathway enrichment analysis

DEGs between the MP7 cluster and all other clusters were identified using the sc.tl.rank_genes_groups function from the Scanpy with the Wilcoxon rank-sum test as the statistical method, and excluded genes with logfoldchanges < 1 and *p* value > 0.01. The resulting DEGs were subsequently subjected to gene set enrichment analysis using the “clusterProfiler” R package (https://github.com/YuLab-SMU/clusterProfiler) [28]. Functional enrichment was performed against the Kyoto Encyclopedia of Genes and Genomes (KEGG) and Gene Ontology (GO) databases via the clusterProfiler::enrichKEGG and clusterProfiler::enrichGO functions, respectively. Significantly enriched pathways and terms were defined as those with an adjusted *p* < 0.05.

### Batch effect removal of bulk-RNA datasets

We adopted the “SVA” package(https://bioconductor.org/packages/release/bioc/html/sva.html) to eliminate batch effects of bulk RNA data [29]. Principal Component Analysis (PCA) was performed using “FactoMineR”(https://cran.r-project.org/web/packages/FactoMineR/index.html) and visualized using the “Factoextra”(https://cran.r-project.org/web//packages/factoextra/index.html) software package.

### Construction and validation of the risk model

The coxph function of the “survival” package(https://github.com/therneau/survival) was used to perform univariate Cox regression analysis, and the prognosis-related genes were identified based on the criteria of *p* < 0.01. We adopted a method that encompasses 101 combinations of machine learning which has been proven to robustly identify key genes for developing reliable hypoxia signatures, including Super Partial Correlation (SuperPC), Random Forest, Support Vector Machine (SVM), Least Absolute Shrinkage and Selection Operator (Lasso), Gradient Boosting Machine (GBM), Elastic Net, Stepwise Cox, Ridge, CoxBoost, and Partial Least Squares with Cox regression (plsRcox). Among them, RSF, LASSO, CoxBoost, StepCox bidirectional, and StepCox reverse were used to perform the first step of dimensionality reduction and variable screening [30]. The TCGA data were split into a training set and an internal validation set, while GSE52903 is considered as the external validation set, and the signature with the minimum number of genes and the best C-index was ultimately selected.

Hypoxia-related risk score(HPRS)=0.9715×EGLN1 + 0.1442×ITGA5 − 0.7106×DAPK2 + 0.2035×PLOD2 + 0.2073×P4HA2 + 0.6229×AGPAT4.

### Evaluating the predictive value of the model

We stratified patients according to the hypoxia signature and determined the optimal cutoff value using the surv_cutpoint function in the “survminer” package(https://github.com/kassambara/survminer). Kaplan-Meier (KM) survival analysis was employed to assess the prognostic impact of the hypoxic signature. Additionally, ROC curves were utilized to validate the accuracy and stability of predicting 1-year, 3-year, and 5-year survival based on the hypoxia signature, which were generated through the “timeROC” package(https://github.com/cran/timeROC/blob/master/R/timeROC_3.R).

### Clinical relevance of the HPRS

We conducted univariate and multivariate Cox regression analysis using the “survival” package to determine whether the hypoxia signature is an independent prognostic factor for CSCC patients, and visualized the results using the “forestplot” package(https://cran.r-project.org/package=forestplot). Based on this, a nomogram of the TCGA cohort was constructed for clinical application including age, T, N, M, pathologic stage, and the hypoxia signature. Furthermore, to validate the association between the hypoxia signature and clinical staging with a larger sample size, in addition to the TCGA and GSE52903 cohorts, we included GSE100080, GSE7410, GSE146114, and GSE26511 cohorts. The relationship between Figo staging and hypoxia signature was visually demonstrated through a boxplot generated by the “ggplot2” package(https://cran.r-project.org/package=ggplot2).

### Immune landscape analyses

The “ESTIMATE” R package(https://estimate.r-forge.r-project.org/) was employed to analyze the stromal, immune, and ESTIMATE score of CSCC patients to quantify immune activation levels based on gene expression profiles. Furthermore, the assessment of the abundance of immune microenvironment and functions was conducted by the “IBOR” package(https://github.com/IOBR/IOBR), which includes several bioinformatics algorithms like “MCP-counter”, “IPS, “CIBERSORT” and “quanTIseq” algorithms [31].

### Tumor mutation burden and drug response analyses

SNP information was collected from the TCGA database, and then the mutation profiles were analyzed based on the risk stratification using the “maftools” R package (https://www.bioconductor.org/packages/release/bioc/html/maftools.html) [32]. We applied the tumor immune dysfunction and exclusion(TIDE) and the Immune phenotype score(IPS) to predict the potential immunotherapy responses in CSCC. IPS results for 20 solid tumors in TCGA are available on the TCIA (https://tcia.at/home) website and we downloaded the results for CSCC, additionally obtaining the TIDE score on the TIDE website (http://tide.dfci.harvard.edu) [33]. In addition, alterations in genomes significantly influence the treatment response and in many instances, are potent biomarkers for the prediction of drug responsiveness. We obtained the information on drug sensitivity in cancer cells from The Genomics of Drug Sensitivity in Cancer (GDSC) database (www.cancerRxgene.org) to explore the correlation between hypoxia signature and drug responses in various cell lines.

### Cell culture

Human cervical squamous-cell lines SiHa and Caski were cultured in DMEM (BasalMedia, China) supplemented with 10% fetal bovine serum (Sijiqing, 13011 − 8611) and 100 U mL⁻¹ penicillin–streptomycin (Servicebio, G4003). Cells were cultured at 37 °C in a humidified atmosphere containing 5% CO₂. The medium was refreshed every 2–3 days. For subculture, cells were detached using 0.25% trypsin-EDTA (Boster Biological Technology, PYG0067 ) at approximately 80–90% confluency and passaged at a ratio of 1:3 to 1:5.

### siRNA-mediated knockdown of P4HA2 in SiHa and Caski cells

Three independent siRNAs targeting P4HA2 mRNA were designed and designated siP4HA2-#1, #2 and #3. All siRNAs were synthesized by Sangon Biotech (Shanghai, China). Eighteen hours prior to transfection, 3 × 10⁵ cells were seeded into 6-well plates so that the monolayer was 50–60% confluent at the time of transfection. siRNA (final concentration 50 nM) and 5 µL Lipofectamine™ 3000 (Invitrogen, L3000001) were each diluted separately in 125 µL Opti-MEM reduced-serum medium (Gibco, 31985070), mixed gently, and incubated at room temperature for 15 min to form complexes. The mixture was added dropwise to cells containing 2.5 mL antibiotic-free complete medium per well. After 6 h, the transfection medium was replaced with fresh complete growth medium. Cells were harvested 48 h post-transfection for downstream analyses.

### Real-time quantitative polymerase chain reaction (qPCR)

Total RNA was extracted from the SiHA and CasKi cells using a column-based purification method with the FastPure Complex Tissue/Cell Total RNA Isolation Kit (Vazyme, RC113-01) following the manufacture’s data sheets. Briefly, we used ABScript III RT Master Mix for qPCR with the gDNA Remover kit (ABcolony, RK20429) to reverse transcribe 1 µg of total RNA to synthesize complementary DNA (cDNA) for subsequent experiments. The qPCR reaction was initiated with a master mix (ABcolonal, RK21220) containing DNA polymerase, deoxynucleotide triphosphates (dNTPs), a SYBR Green dye, template cDNA, and primers (Sangon Bioctech, Shanghai, China).

PCR amplification was performed under the following conditions:

- 37°C for 2 min
- 95°C for 3 min
- 40 cycles of: 95°C for 5s 60°C for 30s

We used the 2^-ΔΔCt method to relatively quantify the target gene and described the changes in gene expression levels using β - actin as a reference gene. Primer sequences: The primers used for amplification were synthesized by Sangon Biotech (Shanghai, China). The sequences are as follows:

- P4HA2 : Forward 5‘-GGCCTGGTTTGGTGTCCTG-3’; Reverse 5‘- GCCCAGCTCTTAATCTTGGAAAG − 3’.
- HIF-1α : Forward 5‘-GAACGTCGAAAAGAAAAGTCTCG-3’; Reverse 5‘- CCTTATCAAGATGCGAACTCACA − 3’.
- β-actin (Reference) : Forward 5‘-CCTGGCACCCAGCACAAT-3’; Reverse 5‘- GGGCCGGACTCGTCATAC-3‘.

### Western blot

We used RIPA lysis buffer to lyse SiHa and CasKi cells, and measured protein concentration using the BCA assay kit (Servicebio, G2026-200T). The final sample volume per well was set at 20 µg. Protein was separated by 10% SDS-PAGE under constant voltage (120 V, 1.5 h), and transferred from gel to polyvinylidene fluoride (PVDF) under 220 mA for 20 min. Then, the membrane was blocked with protein-free rapid blocking buffer (Epizyme, PS108P) at room temperature for 1 h, and incubated overnight with the target protein-specific primary antibody (Proteintech, 13759-1-AP;20960-1-AP) at 4 ° C, followed by washing with TBST. Apply secondary antibody (Abcam, ab205718) at room temperature for 1 h, and then wash further with TBST.

### Transwell migration/invasion assays

Cell migration and invasion assays were performed using Transwell chambers (24-well plate, 8 μm pore size). For the migration assay, 5 × 10^4^ cells in 200 µL of serum-free medium were seeded in the top chambers of Transwell plates with membrane inserts without Matrigel. For the invasion assay, the membrane inserts were precoated with a Matrigel (diluted 1:8 in serum-free DMEM, 60 µL per insert) to a uniform layer on the apical side before 1 × 10^5^ cells in 200 µL of serum-free medium were seeded. 0.5 mL of DMEM supplemented with 10% FBS was added to the lower compartment of the culture plate. The plate was incubated for 24 h to allow cell migration or invasion. Following that, We fixed the cells with 4% paraformaldehyde for 15 min, and then stained them with crystal violet for 30 min.

### CCK-8 assays

Cells in the logarithmic growth phase were seeded into 96-well plates at a density of 2.5 × 10^3^ cells per well in 100 µL of culture medium. After overnight incubation to allow cell attachment, cells were subjected to the indicated treatments and cultured for 48 h. At the time of assay, 10 µL of CCK-8 solution (Vazyme, A311-01) was added to each well, and the plate was incubated for 1–4 h. The absorbance at 450 nm was measured using a microplate reader. The optical density values obtained from the assay were used to determine the number of viable cells and assess cell proliferation or cytotoxicity. The absorbance values were subtracted by the background absorbance from blank wells and normalized to control samples to calculate the percentage of cell survival or inhibition.

### Statistical analysis

We used R 4.3.2 software for data processing, statistical analysis, and visualization, with statistical significance defined as *p* < 0.05.

## Result

### Identification of tumor cell state diversity

**Fig. 1:**
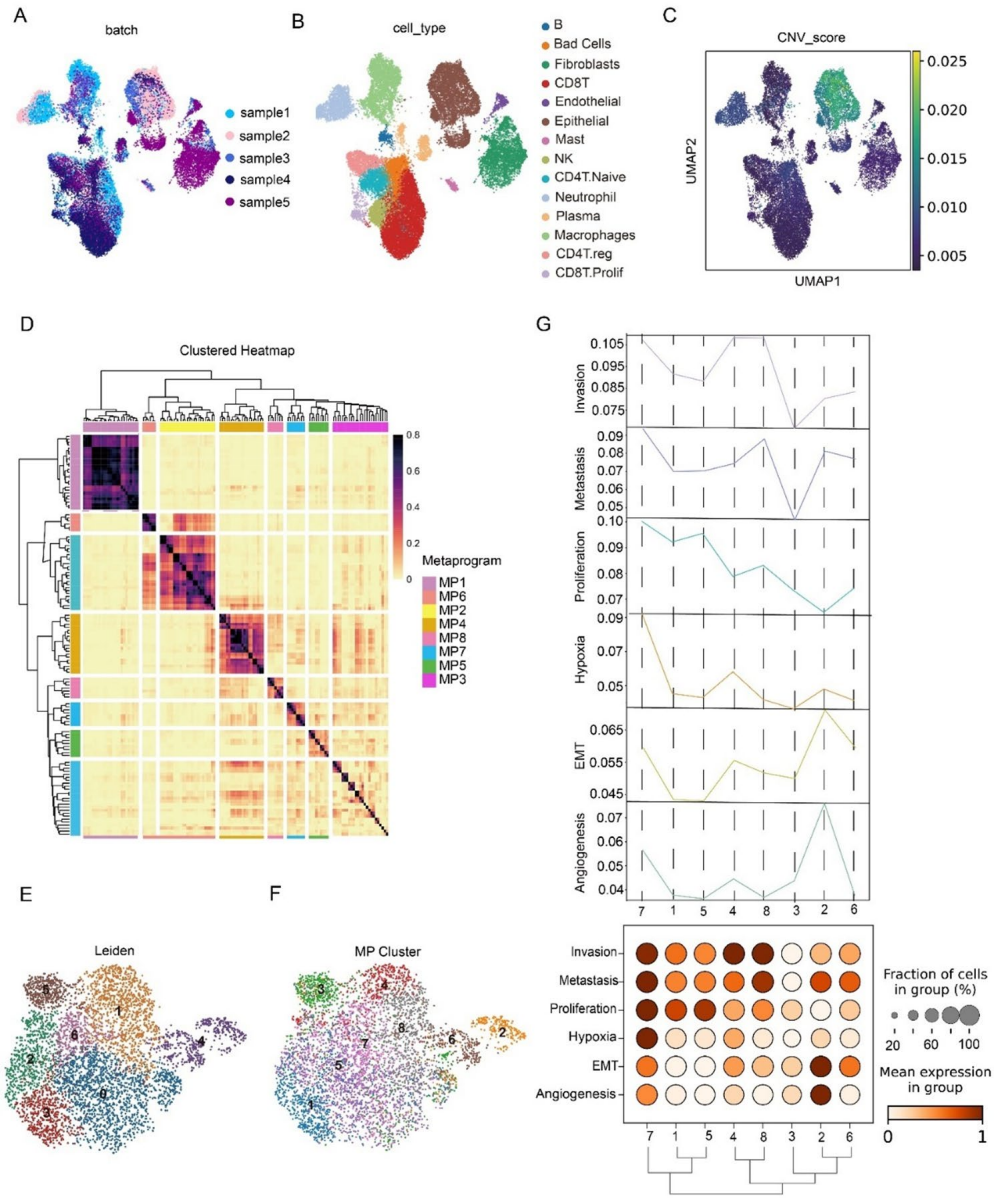
CSCC scRNA-data data analysis displays the characteristics of tumor cell subtypes. **A** Umap plot of integrated scRNA-seq profiles of 5 CSCC patients. The cell color corresponds to the sample. **B** Same Umap plot as (A) but colored by cell clusters. **C** Same Umap plot as (A) but colored by CNV_scores. **D** Identification and clustering of NMF programs in epithelial cells with high copy number variations in CSCC patients. Different colors represent the tumor MPs identified from consensus NMF programs. **E** Umap plot of epithelial cells with high copy number variations in CSCC patients, colored by cell clusters. **F** Same Umap plot as (A) but colored by MPs. **G** The malignant phenotype scores between MPs were displayed through dotplot and line graphs. Below the figure are tumor MPs and their clusters, while on the left are multiple malignant phenotypes to be evaluated

In order to explore the heterogeneity in multiple samples and reveal conserved cellular states of cancer cells in different individuals, we integrated 5 CSCC samples from 2 publicly available cervical cancer datasets in GEO (Fig. 1A). The top 3000 highly variable genes were retained for further analysis, followed by batch effect removal. Visualization using UMAP clustering revealed that 28,537 cells from CSCC were clustered into 24 subgroups (Fig. S1A). These clusters were assigned to distinct cell types based on the expression of canonical marker genes (Fig. S1B). We identified 11 cell types, including B cells, plasma cells, endothelial cells, epithelial cells, Macrophages, neutrophils, NK cells, fibroblasts, mast cells, and T cells (Fig. 1B). Cancer cells were identified with inferCNV (Fig. 1C) [34], and clustered into 7 leidens for further analysis (Fig. 1D). To capture the intrinsic heterogeneity of cancer cells in CSCC and assess their impact on patient prognosis, we applied NMF to identify consensus cellular states shared across individuals. We identified eight meta programs (MPs) (Fig. 1E) and assigned each tumor cell to the MP for which it had the highest expression score, calculated using the top 30 genes of each MP. These assignments were subsequently visualized on a UMAP dimensionality reduction map (Fig. 1F). To comprehensively characterize each MP and explore its associations with aggressive tumor phenotypes, we evaluated seven features widely reported to correlate with poor outcomes in CSCC, including invasion, metastasis, proliferation, angiogenesis, EMT (epithelial-mesenchymal transition), stemness, and hypoxia. MP7 stood out among the seven programs, showing elevated scores in invasion, metastasis, proliferation, hypoxia, and angiogenesis, with hypoxia specifically upregulated only in this cluster and unchanged in other programs. These findings suggest a strong association between MP7 and hypoxia, along with a pronounced malignant phenotype, warranting the need for further in-depth exploration of MP7 (Fig. 1G).

### MP7 is related to cancer aggressiveness in multiple CSCC cohorts

**Fig. 2:**
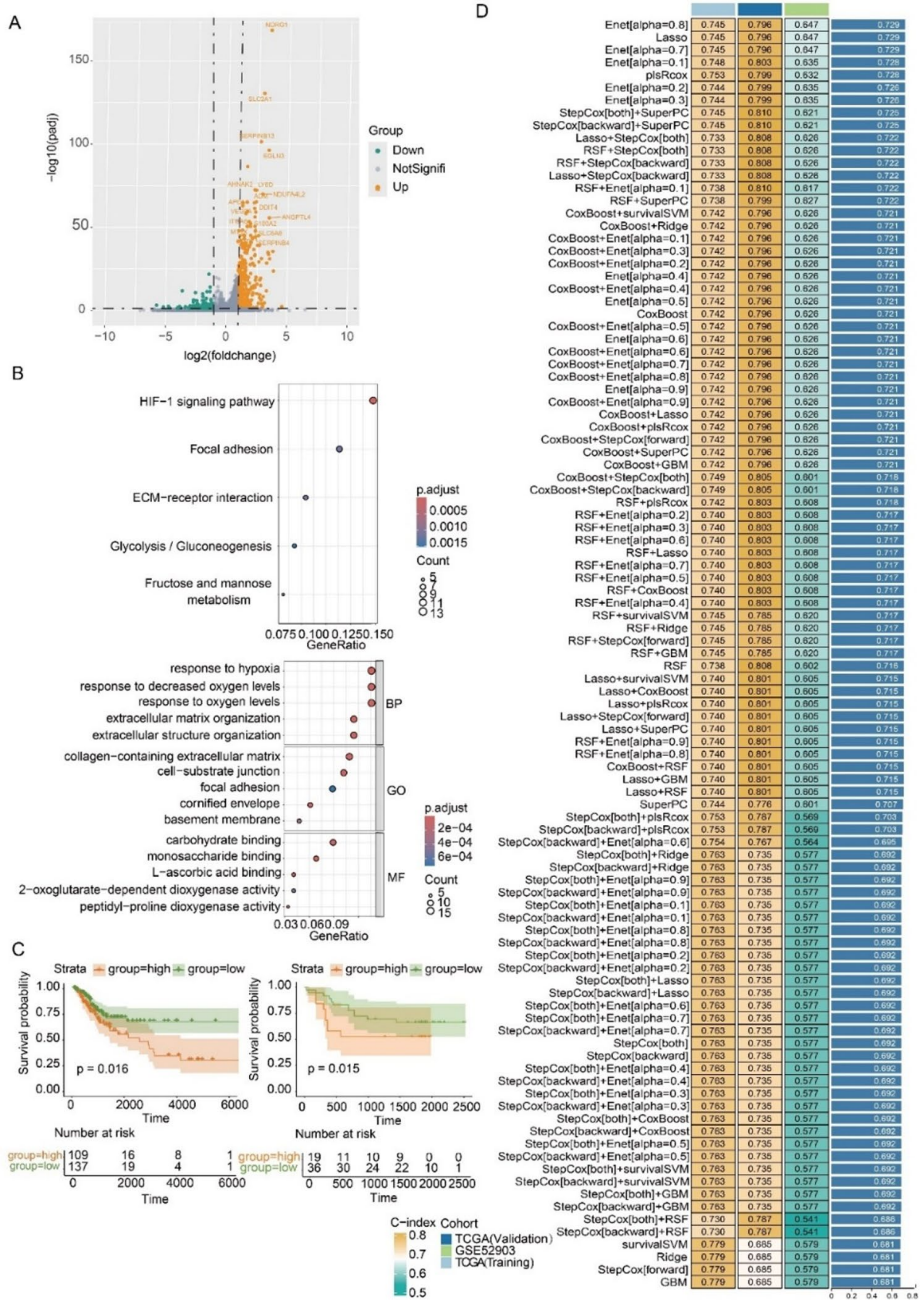
Constructing a hypoxia signature based on hypoxia-related MP7. **A** The volcano map displays the differential genes identified between MP7 and other MPs. **B** Pathway enrichment analysis of HRDEGs between MP7 and other MPs through KEGG and GO. **C** Kaplan Meier analysis demonstrated the association between MP7 and OS in patients in the TCGA and GSE52903 cohorts. **D** Construction of the Hypxia signature based on 101 machine learning combinations. The consistency index in the training cohort, internal validation cohort, and external validation cohort was displayed

To further reveal the biological characteristics of MP7, we identified the differentially expressed genes (DEGs) between MP7 and the other MP clusters, hereafter referred to as Hypoxia Related Differentially Expressed Genes (HRDEGs) (Fig. 2A). HRDEGs with logfoldchanges > 1 and *p value* < 0.01 were included for downstream analysis. Functional enrichment analysis using KEGG and GO demonstrated that HRDEGs are largely associated with hypoxia-related biological processes, especially the HIF-1 signaling pathway, as well as focal adhesion and PI3K-Akt signaling pathway (Fig. 2B), which were reported to be involved in cell proliferation, anti-apoptosis, invasiveness, and metastasis [21, 22, 35, 36].

To validate the correlation between the aggressive tumor features presented by MP7 and the prognosis of CSCC, we conducted survival ·analysis in the TCGA and GSE52903 cohort. Patients were divided into high-expression and low-expression groups based on the top 10 HRDEGs expression levels. In both datasets, the patients with high-expression of this hypoxia-related subtype were demonstrated to have inferior clinical outcomes (Fig. 2C), which suggested that MP7 may be intricately linked to tumor progression and accordingly be correlated with an unfavorable prognosis.

Accordingly, we next aimed to develop a hypoxia related signature derived from MP7 that can effectively predict the prognosis of CSCC patients, thereby facilitating early risk stratification and intervention to improve patient outcomes. To increase the sample size and assess the generalizability of the hypoxia-related signature, we combined the TCGA and GSE52903 datasets with stringent batch effect removal. This approach allowed us to develop a signature in a larger cohort while seeking to reduce potential bias(Fig. S1C). After increasing the sample size, we conducted univariate Cox regression analysis for each HRDEG, revealing 30 genes significantly correlated with CSCC prognosis. (Fig. S1D). We then employed a rigorous selection process using 101 distinct combinations of machine learning methods to systematically identify the most robust ensemble model and the optimal prognostic gene combination from the aforementioned HRDEGs to ensure stable and reliable prediction of CSCC outcomes. Among all candidate signatures, the one showing the highest C-index and comprising the fewest genes in both the training and validation sets was prioritized for further assessment. After filtering the best gene combinations by machine learning, we further refined this selection process by constructing a multivariate Cox regression model, which allowed us to identify the genes that independently contribute to patient prognosis. The “Enet[alpha = 0.8]” combination was recognized for its smaller number of genes yet robust predictive power. (Fig. 2D). We identified the coefficients of the genes included in the “Enet[alpha = 0.8]” combination through multivariate Cox regression and established a hypoxia signature, which was used to calculate the risk score (HPRS) for patients.

### Validation and evaluation of the hypoxia signature in the training and validation set

**Fig. 3:**
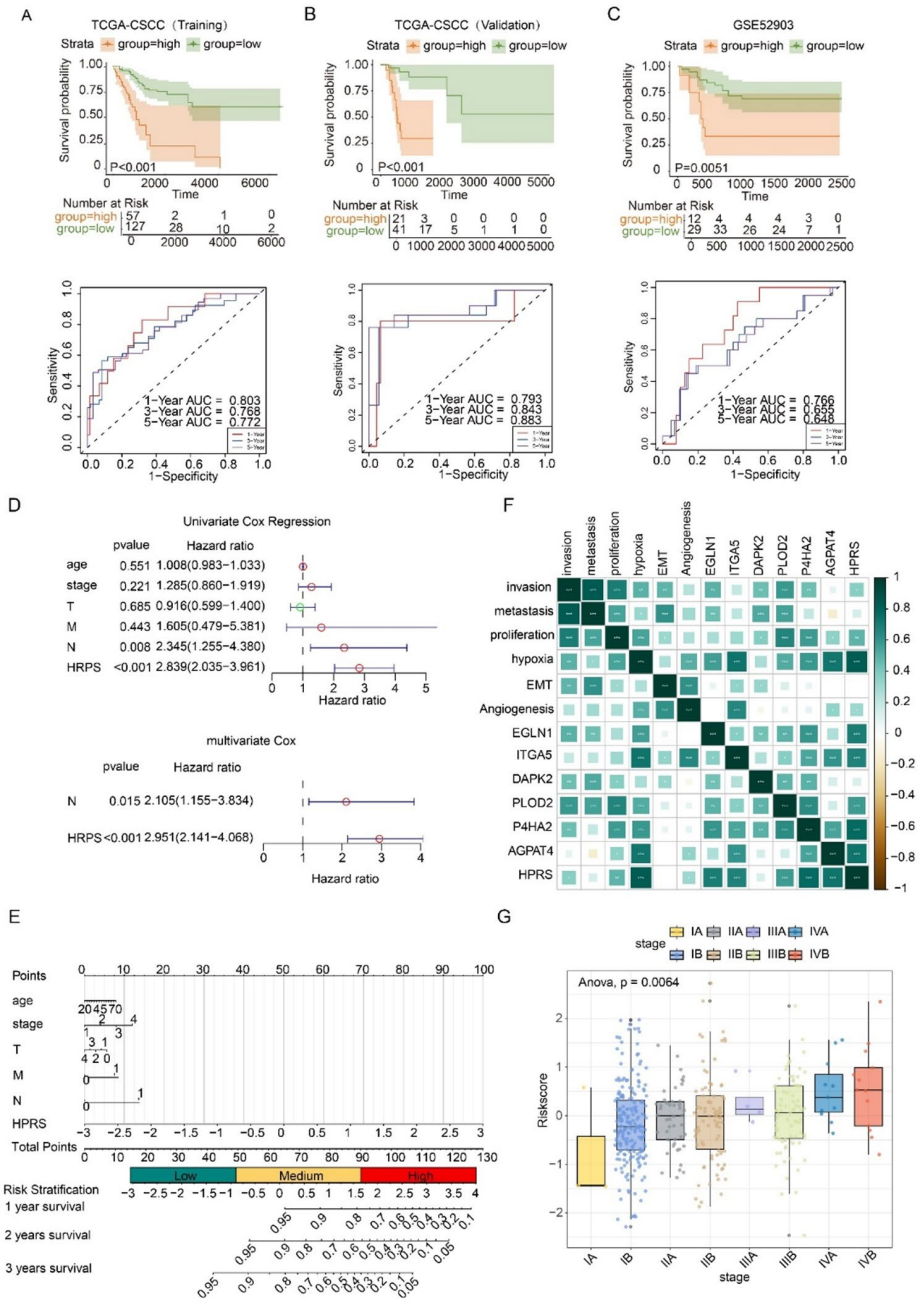
Evaluation of the prognostic potential of hypoxia signature. **A**-**C** The Kaplan–Meier survival curves demonstrated that the hypoxia signature exhibits significant prognostic potential. The ROC curves illustrated the model’s accuracy in predicting 1-year, 3-year, and 5-year survival within both the training and testing datasets. **D** Perform univariate and multivariate Cox regression analysis in the TCGA cohort. **E** The nomogram integrates various clinical and molecular factors to predict the 1-year, 3-year, and 5-year survival probabilities for patients with cervical squamous cell carcinoma (CSCC). Each variable is assigned a specific point value based on its influence on survival outcomes. **F** The correlation heatmap shows the correlation between the Hypoixa signature and various malignant phenotypes(invasion, metastasis, proliferation, hypoxia, EMT, angiogenesis). **G** Box plot of HRPS in different stages (IA, IB, IIA, IIB, IIIA, IIIB, IVB). The results showed a significant correlation between staging and HRPS (p=0.0054)

To evaluate the efficacy of HPRS in performing effective prognostic stratification of patients, the “survminer” package was used to determine the optimal cut-off point to divide patients into HPRS^High^ and HPRS^Low^ groups for survival analysis. Kaplan-Meier (KM) survival analysis implied that patients of the HPRS^high^ group exhibited significantly worse prognosis in comparison with those of the HPRS^low^ group. In addition, the predictive capability of HPRS was evaluated over time spans of 1, 3, and 5 years, demonstrating the area under the ROC curve (AUC) values remained stable within different duration supporting the resilience of HPRS to following periods [37] (Fig. 3A). In the validation cohort and the independent cohort GSE52903, HPRS also showed promising prediction of shorter OS (Fig. 3B-C). To determine whether the hypoxia signature can serve as an independent prognostic factor for CSCC patients compared to other clinical features, we conducted univariate and multivariate Cox analysis. Univariate Cox regression analysis showed that the hypoxia signature and lymph node metastasis were statistically significant related with poor outcomes, while multivariate analysis showed that they were both independent prognostic factors (Fig. 3D). We also developed nomograms that take into account the hypoxia signature and other clinical features (Fig. 3E). This result visually demonstrates the relationship between multiple features and the clinical outcome, showing how each variable’s value affects the predicted outcome, with the degree of impact proportional to the multivariable Cox regression coefficients. Among them, the hypoxia signature accounted for a larger proportion, intuitively demonstrating its high predictive potential. Taken together, these results support the prognostic value of the hypoxia signature and its consistency across independent cohorts.

As a bulk-level signature derived from HRDEGs, the HPRS was further validated in scRNA-seq data to confirm that its biological associations are preserved at the single-cell level. We observed that HPRS was highly correlated with aggressive tumor phenotypes across multiple MPs, faithfully reflecting key aspects of the hypoxia signature, including hypoxia, invasion, angiogenesis, and proliferation (Fig. 3F). Furthermore, to extend the prognostic relevance of HPRS, we evaluated its association with FIGO clinical staging across CSCC patients. We included the GSE100080, GSE7410, GSE26511, and GSE146114 datasets in addition to the original training and validation datasets (TCGA and GSE52903) and found that a higher HPRS is significantly associated with more advanced FIGO stages. This is consistent with the results of the previous multivariate Cox regression analysis, which indicated that tumor advanced staging was not an independent risk factor compared to HPRS. This indicates that HPRS is a more central driver of the observed prognostic relationship (Fig. 3G). 

### Comparison of tumor immune landscape between high- and low-HPRS groups

Hypoxia has a profound impact on the biological behavior and aggressive phenotype of cancer cells, as well as disturbance in the immune landscape in the tumor microenvironment [38–40]. It is proven that hypoxia is involved in tumor immune escape and promotes tumorigenesis [41]. Therefore, we aimed to investigate the potential association between HPRS and immune infiltration.

**Fig. 4:**
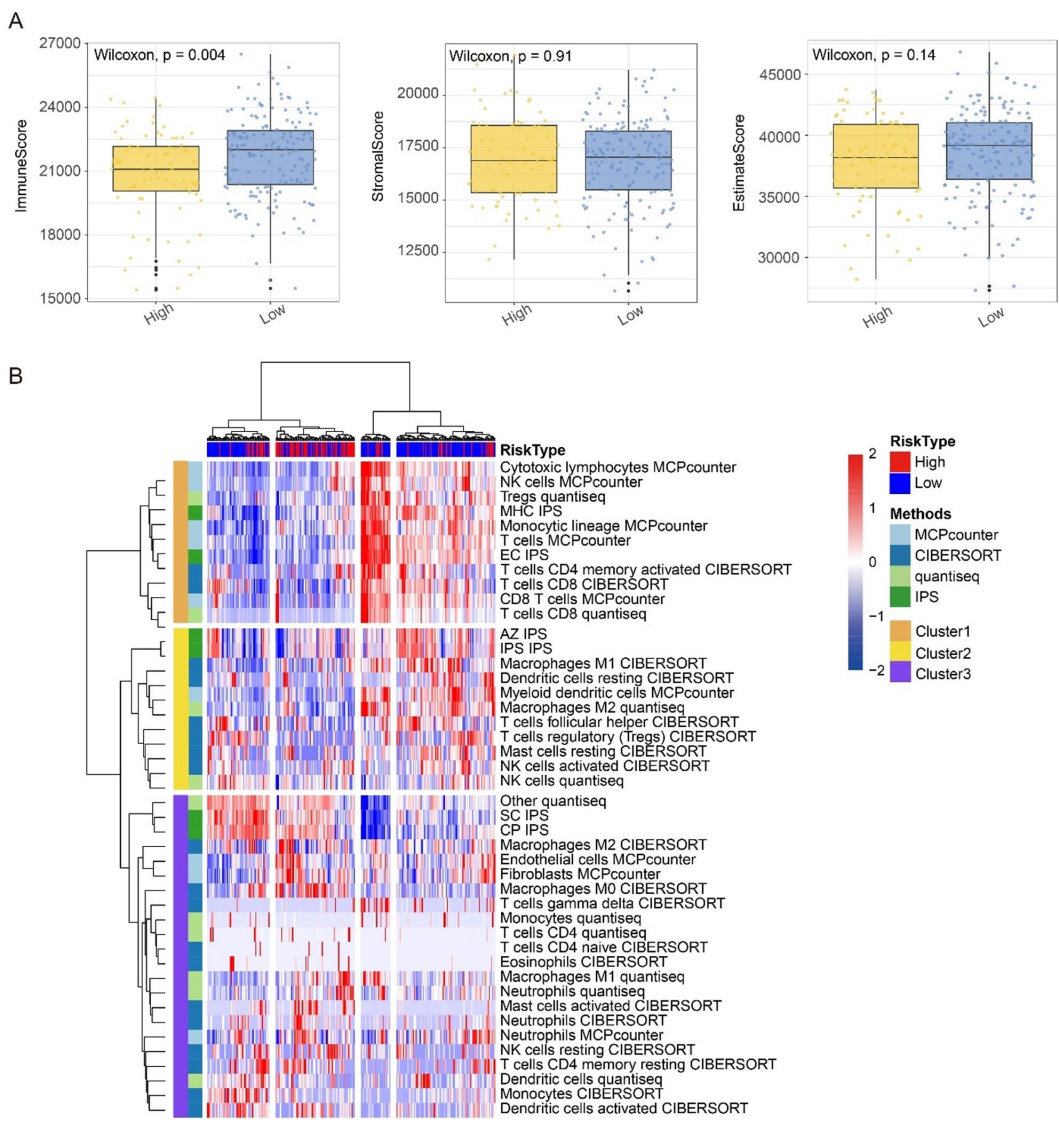
Comparison of tumor microenvironment across HRPS groups. **A** The box plot displays the ESTIMATE score, Immune score, and Stroma score between the HRPSHigh and HRPSLow groups. The p value was given. **B** The heatmap illustrates the distinct immune infiltration patterns between the HRPSHigh and HRPSLow groups, as characterized through comprehensive analysis using multiple computational approaches

To demonstrate the distinct immune microenvironments between HPRS^high^ and HPRS^low^ groups, we initially used ESTIMATE to assess the stromal and immune scores of the patients. The ESTIMATE algorithm quantifies tumor microenvironment characteristics through three distinct indices: (i)the immune score, which evaluates the level of immune cell infiltration; (ii)the stromal score, reflecting the proportion of fibroblasts and endothelial cells; (iii) the ESTIMATE score is the sum of the previous two, which inversely correlates with tumor purity. In our cohort, tumors with high HPRS exhibited a significantly elevated immune score, suggesting a greater abundance of immune cells. In contrast, no statistically significant differences were observed in stromal or ESTIMATE scores between high- and low-HPRS tumors, indicating that stromal composition and overall tumor purity were comparable across the two groups (Fig. 4A). We further employed four different algorithms, including the MCP counter, CIBERSORT, IPS, and quanTIseq, to evaluate the distinct immune activation and related biological processes. We excluded B cells from the immune cell infiltration assessment, given the inconsistent variation of B cell infiltration deduced among different algorithms (Fig. S2A). Similarly, the HPRS^low^ group showed better immune infiltration. We observed that patients in the HPRS^high^ group were clustered and exhibited a downregulation of pro-immune features, including NK cells involved in innate immunity and DC cells involved in antigen presentation processes. CD8 T cells and activated CD4 cells involved in specific immunity were also downregulated, especially cytotoxic lymphocytes, which secrete various cytokines and play an important role in tumor killing. Meanwhile, an upregulation of immunosuppressive features, such as M2 macrophages and immune checkpoints, was also observed. On the contrary, patients in the HPRS^low^ group were clustered into three categories, among which there were two clusters that showed upregulation of pro-immune features, while one cluster exhibited an immune phenotype similar to that of the HPRS^low^ group (Fig. 4B).

### Predictive value of hypoxia signature for immunotherapy

The Immunophenoscore (IPS) quantifies the expression of immune-related genes to evaluate the immunocompetence of the tumor microenvironment and predict patient responses to immune checkpoint inhibitors [42]. In parallel, the TIDE analysis integrates four key parameters to assess tumor immune evasion potential: (i) the TIDE score, a comprehensive indicator where higher values indicate greater overall immune escape risk and poorer outcomes after immune checkpoint blockade [43, 44]; (ii) the T cell dysfunction score, reflecting the extent of intratumoral T cell exhaustion, with elevated values indicating more impaired cytotoxic function; (iii) the immune exclusion score, which quantifies physical and chemical barriers impeding T cell infiltration, where higher scores correspond to a more pronounced “cold tumor” phenotype; (iv) the MSI score, which is elevated in tumors with microsatellite instability-high (MSI-H) status, a context generally associated with high mutation burden and immunotherapy sensitivity.

Given that hypoxia has been previously reported to contribute to immunotherapy resistance [45] and considering the significant correlation between HPRS and the immune landscape, we further assessed the predictive value of HPRS stratification for immunotherapy outcomes by employing Immunophenoscore (IPS) and Tumor Immune Dysfunction and Exclusion (TIDE) analyses.

**Fig. 5:**
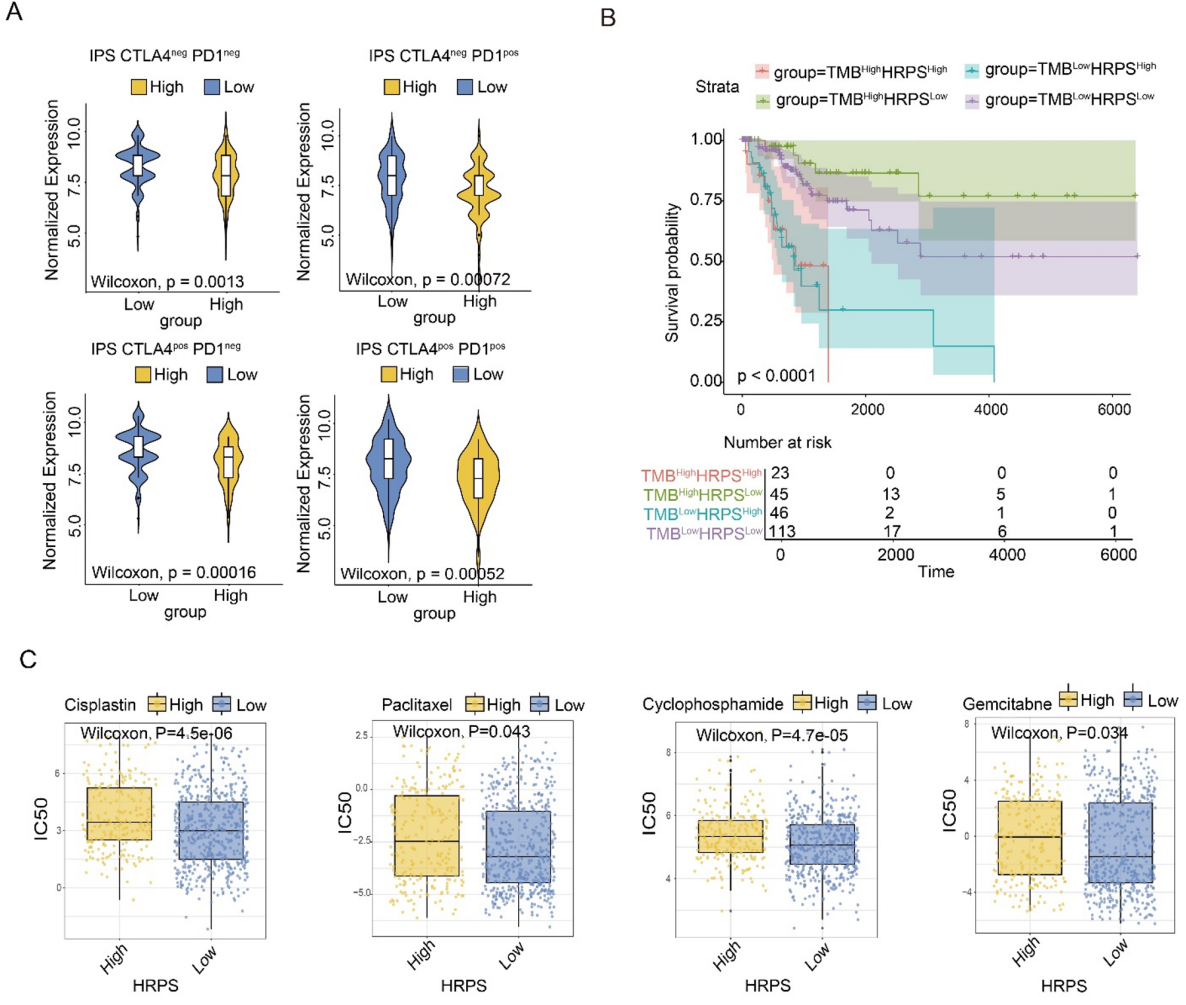
Predictive value of hypoxia signature in immunotherapy. **A** Viollin plot presents the differences in the IPS scores between the two risk groups stratified by CTLA4 and PD-1 treatment. **B** The Kaplan–Meier curve shows the survival differences between different TMB and HPRS groups. **C** Comparison of the IC50 values of chemotherapy between two risk groups, including Cisplatin, Paclitaxel, Cyclophosphamide, and Gemcitabine

In our cohort, the HPRS^high^ group exhibited significantly lower IPS values for anti-PD-1, anti-CTLA-4, and combination therapies, suggesting potentially diminished response to immune checkpoint blockade (Fig. 5A). Correspondingly, TIDE analysis revealed that HPRS^high^ tumors had significantly elevated TIDE and immune exclusion scores, whereas T cell dysfunction and MSI scores were lower (Fig. S2B). Together, these results suggested that HPRS^high^ tumors exhibited worse response to immunotherapy compared with HPRS^low^ tumors, and may foster a “T cell-absent” tumor microenvironment, which may underlie their inferior response to immunotherapy.

TMB may drive a potent anti-tumor immune response, leading to a sustained clinical response to immunotherapy [46, 47]. To understand the tumor mutational burden (TMB) between different HPRS groups and its correlation with HPRS, we used waterfall charts to visualize the somatic mutation maps of the HPRS^High^ and HPRS^Low^ groups. The total mutational burden in the HPRS^high^ group was 81.16%, while that in the HPRS^low^ group was 88.61% (Fig. S2D). However, no statistically significant difference in TMB was observed between the HPRS^high^and HPRS^low^ groups (Fig. S2F). Notably, the HPRS subgroups exhibited significant prognostic differences in both the low and high TMB status subgroups, suggesting that HPRS can effectively stratify patients regardless of their TMB status (Fig. 5B).

### Predictive value of hypoxia signature for chemotherapy

Hypoxia creates favorable conditions for a dominant resistance to multiple antitumor treatments, it may also lead to intrinsic or acquired resistance to chemotherapy [48–50]. We then assessed the relationship between HPRS and chemotherapy response. We interrogated the GDSC database of cancer cell lines and evaluated whether HPRS could effectively stratify the response of CSCC to chemotherapy [51]. The cell lines were divided into the HPRS^high^ group and the HPRS^low^ group as indicated above. We compared the IC50 values of common first-line chemotherapy drugs for cervical cancer between the two groups. It was observed that the IC50 of cisplatin, paclitaxel, cyclophosphamide, and gemcitabine in the HPRS^low^ group was lower than that of the HPRS^high^ group (Fig. 5C). This suggests that the signature has predictive value for chemotherapy response.

### P4HA2 mediates tumor hypoxia tolerance and malignant phenotype

**Fig. 6:**
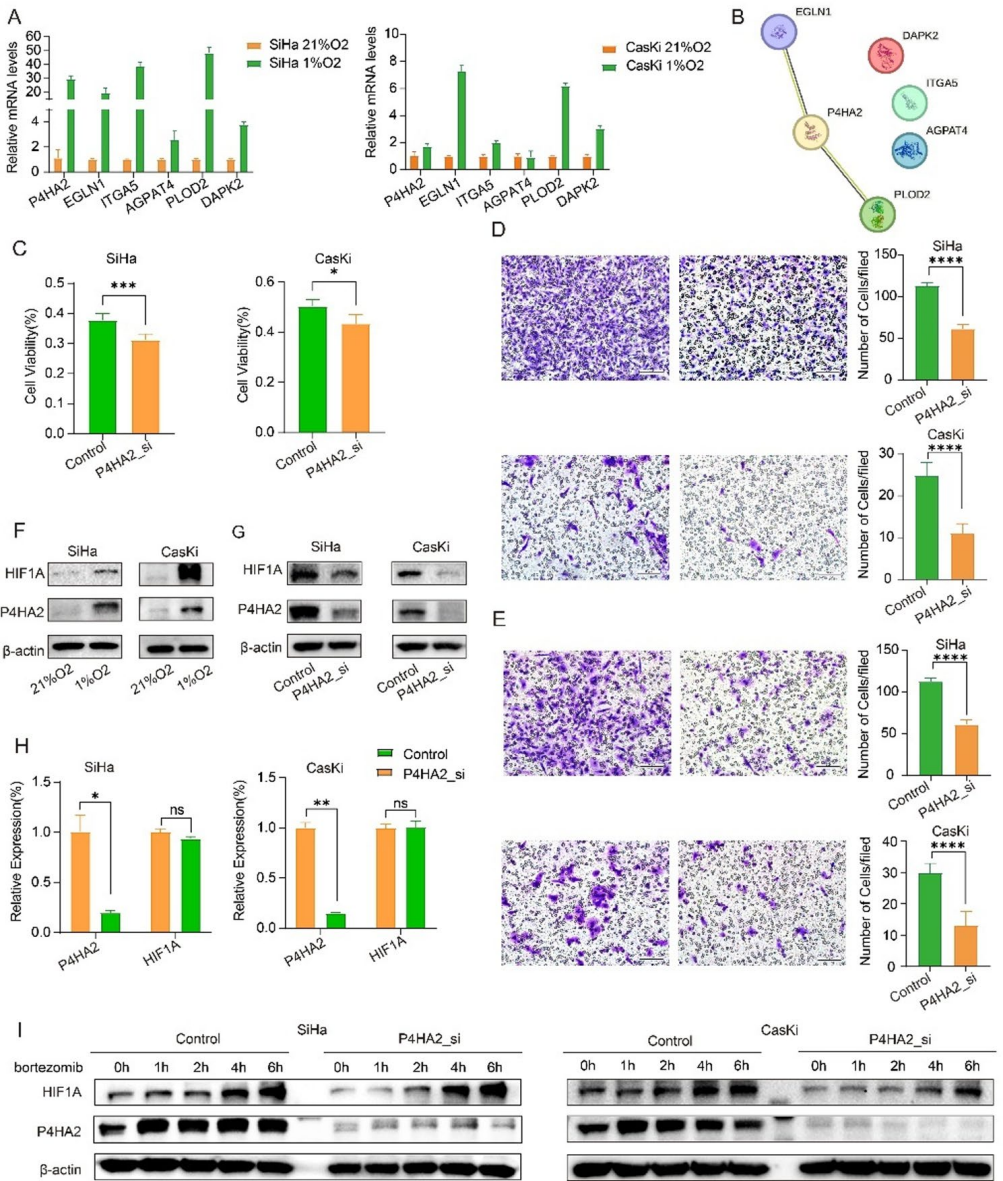
Verification of the expression levels of P4HA2 and its correlation with malignant phenotype. **A** The genes included in the hypoxic signature were quantified by qPCR in SiHa and CasKi cell lines under hypoxic (1% O2) and normoxic (21% O2) conditions. **B** The protein-protein interaction network (PPI) showed the interaction relationship between the genes included in the hypoxic signature. **C** The relative cell viability of control cells and P4HA2 knockdown cells under hypoxic conditions compared to normoxic conditions.* p < .05,**p < .01,***p < .001,****p < .0001. **D** Transwell migration experiment explores the effect of P4HA2 knockdown on tumor cell invasion ability. **E** Transwell invasion experiment explores the effect of P4HA2 knockdown on tumor cell metastasis ability. **F** Western blot results of HIF-1α protein expression in P4HA2 knockdown and untreated groups of SiHa and CasKi cells. **G** mRNA expression of HIF1A in SiHa and CasKi cells in P4HA2 knockdown and untreated groups of SiHa and CasKi cells. **H** Quantitative analysis of relative expression of P4HA2 and HIF1A in Siha and CasKi cell lines after P4HA2 knockdown. **I** Western blot results of HIF-1α protein expression in SiHa and CasKi cells treated with proteasome inhibitors for 0.1.2.4.6 hours after P4HA2 knockdown and control treatment for 48 hours

With the clinical significance of the model established, we validated the expression of HPRS genes under hypoxic conditions in vitro and explored their functional roles. qPCR results demonstrated that hypoxia markedly upregulated these genes in cervical cancer cell lines, corroborating their strong link to hypoxia. (Fig. 6A).

Among these genes, P4HA2 exhibited a significant correlation with the other five in both correlation analysis and occupied a kernel position in protein-protein interaction (PPI) networks, suggesting that P4HA2 may play a critical role in HPRS and indicate its potential as a key regulator in the hypoxia pathway (Figs. 3F and 6B).

We assessed the impact of P4HA2 knockdown under hypoxic conditions on cell migration and invasion by plating SiHa and CaSki cells in the upper chambers of Transwell plates with and without Matrigel coating. Under the stress of hypoxia, the migration and invasion abilities of these two cell lines were significantly impaired due to P4HA2 knockdown (Fig. 6D, E). Additionally, to confirm the essential role of P4HA2 in surviving hypoxic tumor conditions, we knocked down P4HA2 expression via siRNA transfection and found that knockdown of P4HA2 led to a reduction in cell viability after incubated under hypoxic conditions for 48 h compared to normoxic conditions in both SiHa and Caski cervical cancer cell lines, suggesting that P4HA2 downregulation impairs hypoxic tolerance (Fig. 6C).

HIF-1α plays a pivotal role in enabling cells to survive and adapt to hypoxic conditions by regulating a multitude of mechanisms, including angiogenesis, metabolic adaptation, apoptosis, and cell cycle control. As a key molecular regulator of hypoxia, HIF-1α orchestrates these processes to ensure cellular homeostasis and promote cancer survival in low-oxygen environments [52, 53]. To further reveal the mechanism by which P4HA2 modulates the hypoxia tolerance and the transformation of malignant phenotypes in tumor cells, we validated the engagement of P4HA2 in regulating the hypoxia-associated pivotal molecule HIF-1α at the protein level (Fig. 6F). Under hypoxic conditions, we observed a responsive increase in both P4HA2 and HIF-1α protein levels. Additionally, after P4HA2 knockdown, we observed that HIF-1α levels decreased concomitantly with the downregulation of P4HA2, indicating an association between HIF-1α mediation and P4HA2 expression (Fig. 6G). To further investigate whether P4HA2 affects HIF-1α at the transcriptional level, we quantified HIF1A mRNA levels following P4HA2 knockdown. However, no significant changes were detected, suggesting that P4HA2 modulated HIF-1α expression via post-transcriptional modification (Fig. 6H). Previous studies have proved that P4HA2 promotes erdafitinib resistance by regulating the stability of HIF-1α in bladder cancer with FGFR3 alteration [54]. It is widely recognized that HIF-1α undergoes hydroxylation at Pro402 and Pro564 by prolyl hydroxylases (PHDs), which facilitates its binding to von Hippel-Lindau protein (pVHL). pVHL then functions as an E3 ubiquitin ligase, promoting the ubiquitination and subsequent rapid degradation of HIF-1α via the 26 S proteasome pathway. Thus, we employed the proteasome inhibitor bortezomib to block HIF-1α protein degradation [55]. After 48 h of P4HA2 knockdown or control treatment, cells were treated with a proteasome inhibitor to inhibit the degradation of HIF-1α. Subsequently, HIF-1α protein levels were measured at 0, 1, 2, 4, and 6 h post-inhibitor administration. In both groups, accumulation of HIF-1α was observed, indicating that the proteasome inhibitor effectively rescued the reduction in HIF-1α protein levels induced by P4HA2 knockdown (Fig. 6I). These results suggest that P4HA2 contributes to the stabilization of HIF-1α and protects it from degradation in cervical cancer cells.

Together, these data demonstrated the essential role of P4HA2 in stabilizing HIF-1α and subsequently mediating aggressive transformation while hypoxia adaptation in vitro.

## Discussion

Through an integrated analysis of single-cell transcriptomic data and bulk RNA-seq data, our study has identified a hypoxia signature with notable prognostic potential. Although current clinical practice already incorporates certain stratification strategies for CSCC, primarily based on clinicopathological features such as tumor stage, histological type, and lymph node status, these factors fail to capture the extensive phenotypic heterogeneity and transcriptional plasticity inherent in tumor evolution [56, 57]. These challenges highlight the need for biologically grounded frameworks that better reflect the underlying tumor biology.

For decades, it has been proven that hypoxia is a critical factor in cervical cancer progression, contributing to tumor invasion, immune evasion, and resistance to chemotherapy and radiotherapy [49, 58–60]. Previous studies have recognized the potential of hypoxia in predicting tumor prognosis and have reported the development of hypoxia-related gene signatures derived from bulk RNA sequencing data, which demonstrate promising risk stratification capabilities, these approaches may overlook the heterogeneity of the tumor cells [61, 62]. Compared to conventional Bulk-RNA analysis, NMF, as a high-throughput unsupervised method, has been applied to address gene expression commonality among different cells, which overcomes the initial variation of either spatial or temporal conditions [63]. In this study, we applied NMF analysis and identified a distinct cluster, MP7, which exhibited prominent hypoxic characteristics. This cluster was further demonstrated to be strongly associated with multiple malignant phenotypes and poor prognosis. Subsequently, a hypoxia signature constructed based on hypoxia-related differentially expressed genes (HRDEGs) from MP7 proved to steadily stratify patient prognosis in both the training set and multiple validation cohorts.

P4HA2, here in the study, was identified as a key gene in our hypoxia signature. Although P4HA2 has been implicated in extracellular matrix remodeling and resistance to therapy in various cancers, most previous studies relied on hypothesis-driven designs [64–66]. In contrast, we used a high-throughput, data-driven clustering approach that uncovered P4HA2’s previously unrecognized role in CSCC in an unbiased manner. Further experimental validation demonstrated that P4HA2 promotes the survival and malignant phenotype of SiHa and CasKi cells under hypoxic conditions by inhibiting HIF-1α degradation. These results not only provide insight into the mechanistic basis of our prognostic model but also reinforce its robustness and predictive reliability of CSCC prognosis [54, 67–69]. Together with recent efforts to develop P4HA2-targeted therapeutics, these findings collectively emphasize the pivotal role of P4HA2 in cancer progression and its potential as a tractable target in CSCC [70]. Importantly, our study offers potential mechanistic insights that may help guide more precise strategies for its targeting.

### Limitations

While our study represents a preliminary exploration, it also opens several avenues for future research. Validation in prospective cohorts will be crucial to confirm the robustness of our hypoxia signature, especially given the relatively small sample size of the current analysis. Integrating additional factors such as HPV status and smoking history could further enhance the precision of cervical cancer prognosis. Importantly, our findings provide initial evidence that P4HA2 regulates HIF-1α and contributes to malignant phenotypes, highlighting P4HA2 as a promising candidate for therapeutic targeting. A major limitation of our study is that, while we identified the regulatory role of P4HA2 on HIF-1α and its impact on malignant phenotypes, we have not yet developed or evaluated an effective therapeutic strategy targeting P4HA2, which should be addressed in future investigations.

## Conclusions

In this study, by combining scRNA-seq and bulk RNA-seq data, we analyzed the transcriptomic landscape of CSCC. Utilizing machine learning combinations, we established a hypoxia signature as an independent prognostic factor closely related to the patient’s poor outcomes involving an immune suppression profile and therapeutic resistance. Further investigation revealed that the key gene, P4HA2, enhanced HIF-1α stability and drove hypoxia adaptation and malignant progression in CSCC cells in vitro. These insights offer a potential framework for early risk stratification in CSCC patients.

## Supplementary Information

Supplementary Material 1.

Supplementary Material 2.

## Data Availability

The data that support the findings include: scRNA-seq data (GSE208653 and GSE197461) and bulk RNA data(GSE52903, GSE100080, GSE7410, GSE26511, and GSE146114) are available from GEO (https:// www.ncbi.nlm.nih.gov/geo/ ), and TCGA-CESC data are available from TCGA( https://portal.gdc.cancer.gov ). Data are available upon reasonable request.

## Abbreviation

CSCC: Cervical squamous cell carcinoma
GEO: Comprehensive Gene Expression Database
HRDEGs: Hypoxia-related cluster differentially expressed genes
TCGA: Cancer Genome Atlas
CC: Cervical cancer
CCRT: Concurrent chemoradiotherapy
NACT: Neoadjuvant chemotherapy
CNV: Copy number variation
scRNA-seq: single-cell RNA sequencin
NMF: Non-negative matrix factorization
MPs: Metaprograms
KEGG: Kyoto Encyclopedia of Genes and Genomes
GO: Gene Ontology
PCA: Principal Component Analysis
SuperPC: Super Partial Correlation
SVM: Random Forest, Support Vector Machine
Lasso: Least Absolute Shrinkage and Selection Operator
GBM: Gradient Boosting Machine
KM: Kaplan-Meier
TIDE: Tumor immune dysfunction and exclusion
IPS: Immune phenotype score
GDSC: Genomics of Drug Sensitivity in Cance
cDNA: complementary DN
dNTPs: deoxynucleotide triphosphates
EMT: Epithelial-Mesenchymal Transition
AUC: area under the ROC curve
HPRS: Hypoxia-related risk score
HIFs: Hypoxia-inducible factors
TMB: Tumor mutational burden
PHDs: Prolyl hydroxylases
PPI: Protein-protein interaction
HPV: Human papillomavirus
TME: Tumor microenvironment
pVHL: von Hippel-Lindau protein
