# Supplementary figures and images for: A P4HA2 hypoxia signature derived from single cell atlas stratifies conserved subtypes with prognostic significance in cervical squamous cell carcinoma
Source: BMC Cancer. 2026 Jan 21;26:257. doi: 10.1186/s12885-026-15597-z (PMC12910786; doi:10.1186/s12885-026-15597-z)

**Fig.6F**

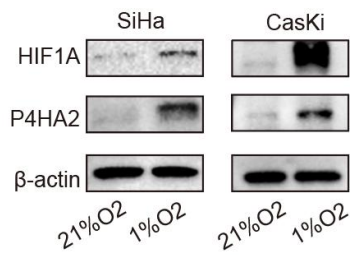

**Fig.6F (Left)**

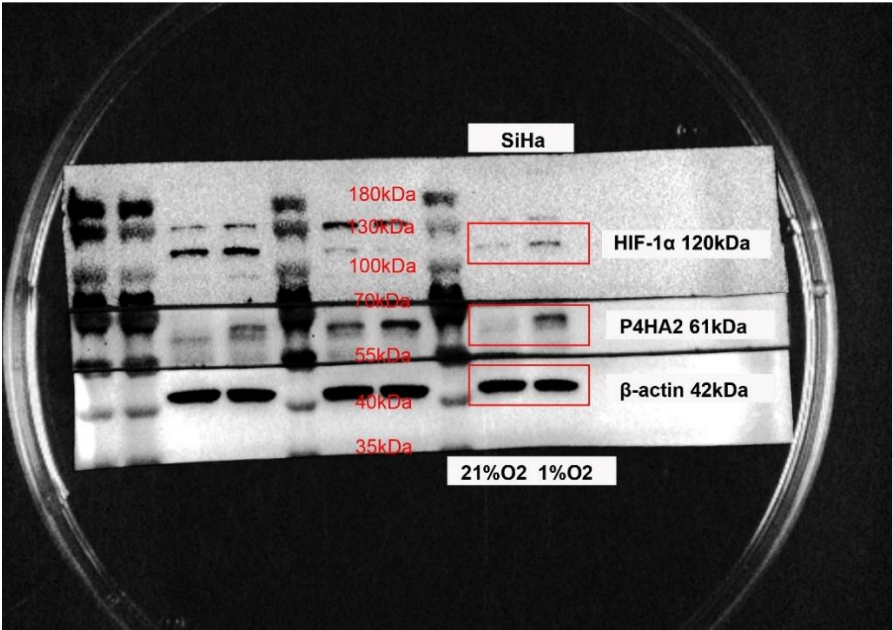

**Fig.6F (Right)**

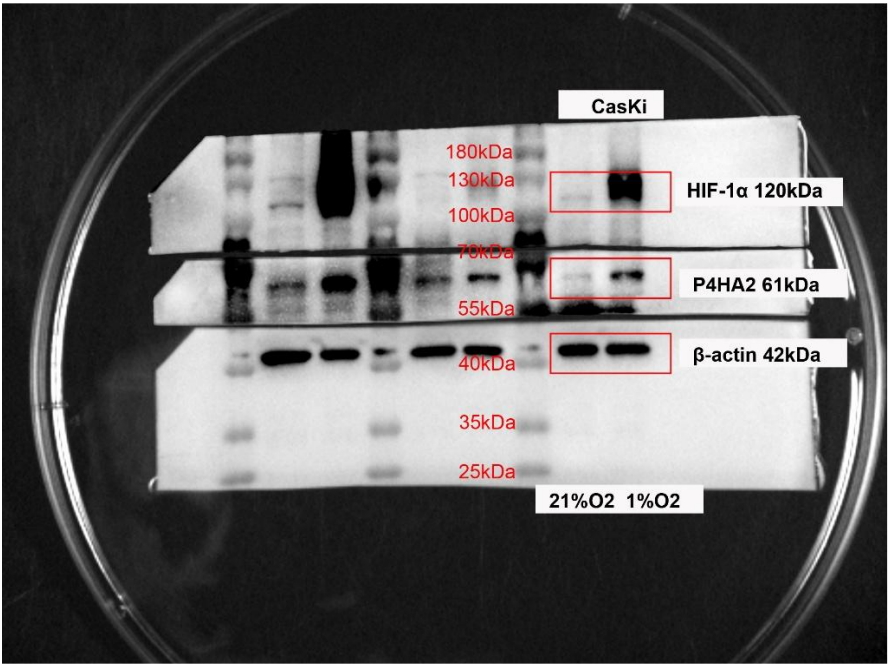

**Fig.6G**

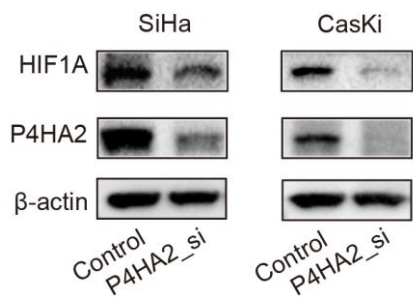

**fig.6G (Left)**

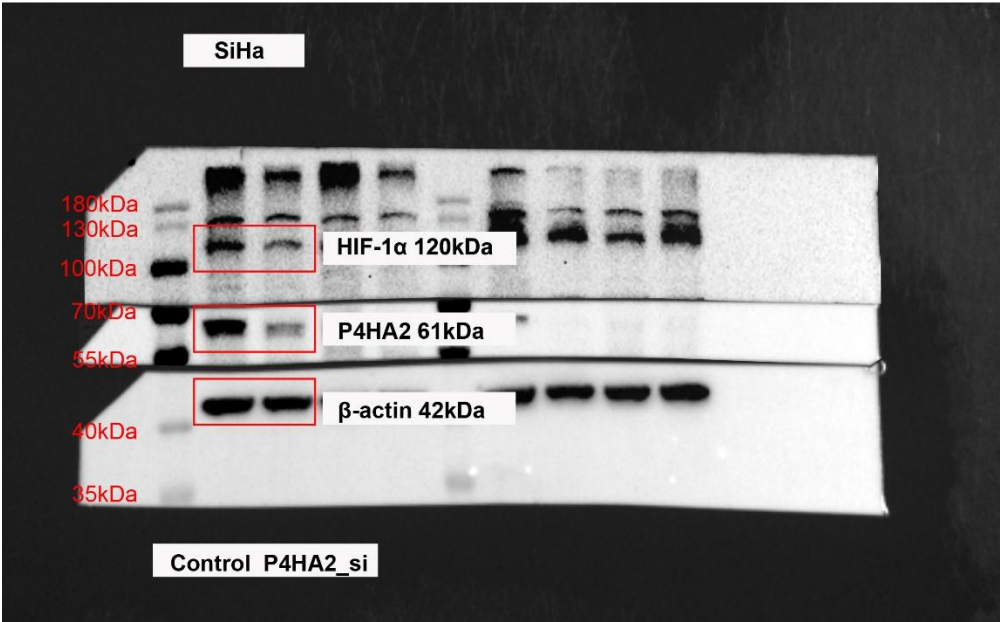

**fig.6G (Right)**

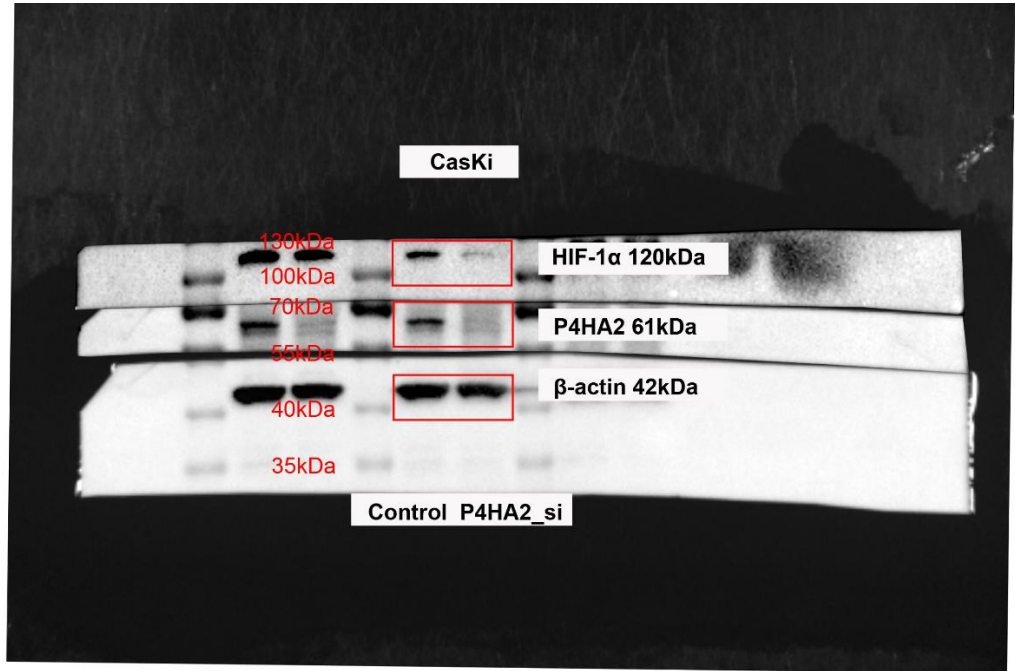

Fig.6I

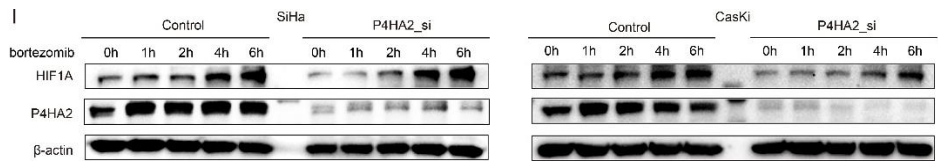

Fig.6I (Left)

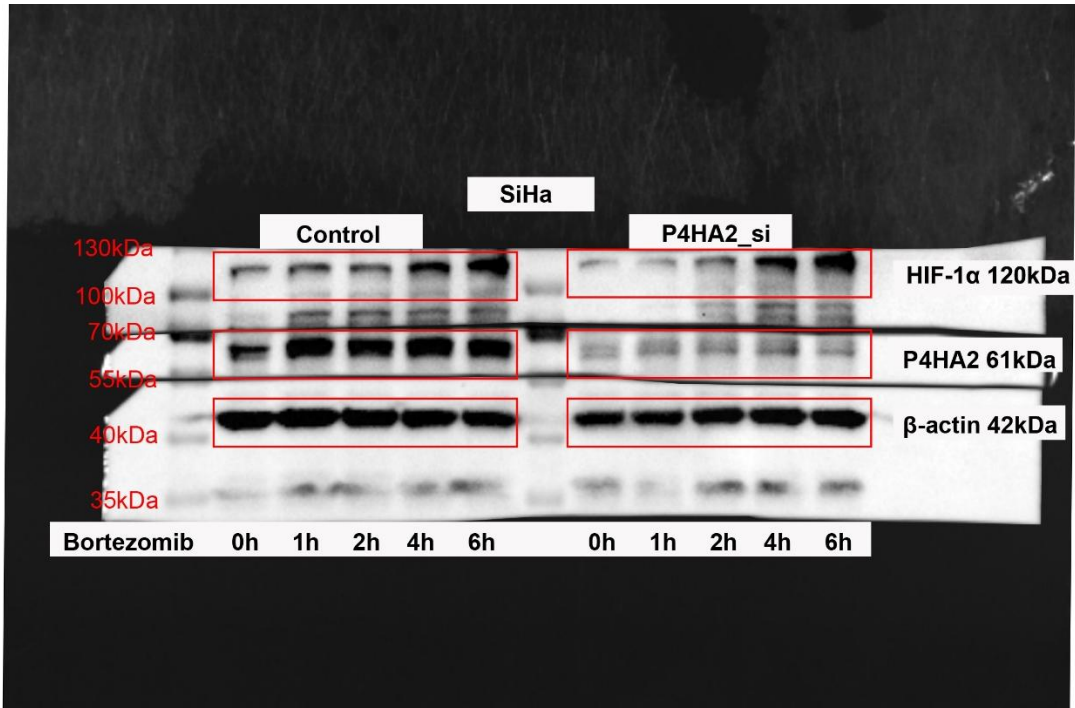

Fig.6I (Right)

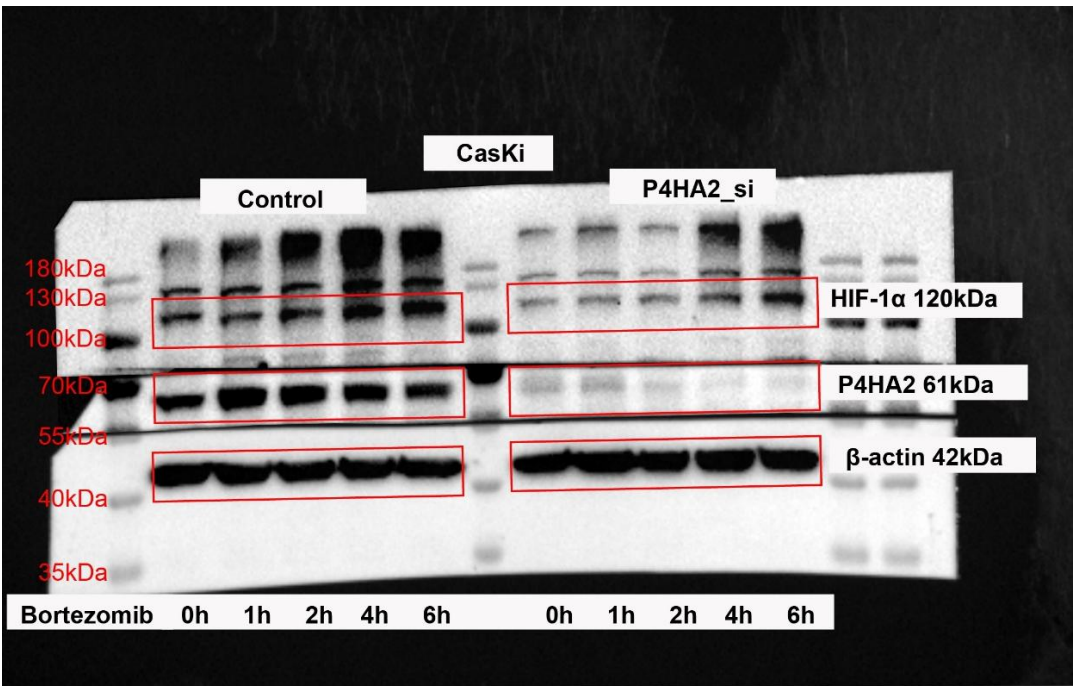

Supplement: Supplementary file 1 — Supplementary Material 1. [file 12885_2026_15597_MOESM1_ESM.pdf]
