# Supplementary material for: A P4HA2 hypoxia signature derived from single cell atlas stratifies conserved subtypes with prognostic significance in cervical squamous cell carcinoma
Source: BMC Cancer. 2026 Jan 21;26:257. doi: 10.1186/s12885-026-15597-z (PMC12910786; doi:10.1186/s12885-026-15597-z)
Supplement: Supplementary file 2 — Supplementary Material 2. [file 12885_2026_15597_MOESM2_ESM.docx]

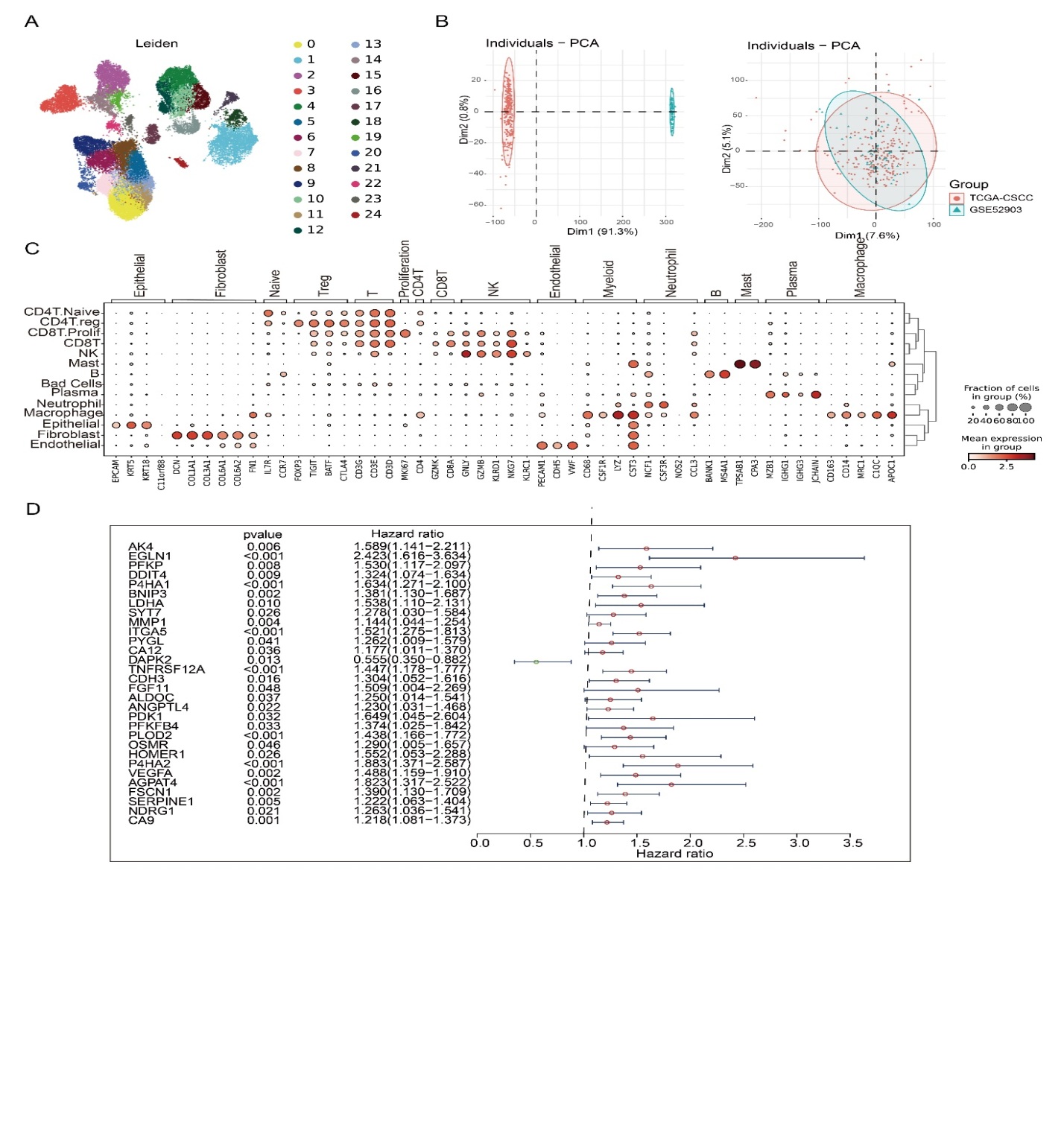


Figure S1

A Umap plot of 24 cell clusters.

B Integrate bulk RNA data and remove batch effect.

C Identify 14 cell types through gene labeling.

D Univariate Cox regression identified HRDEGs with prognostic ability.


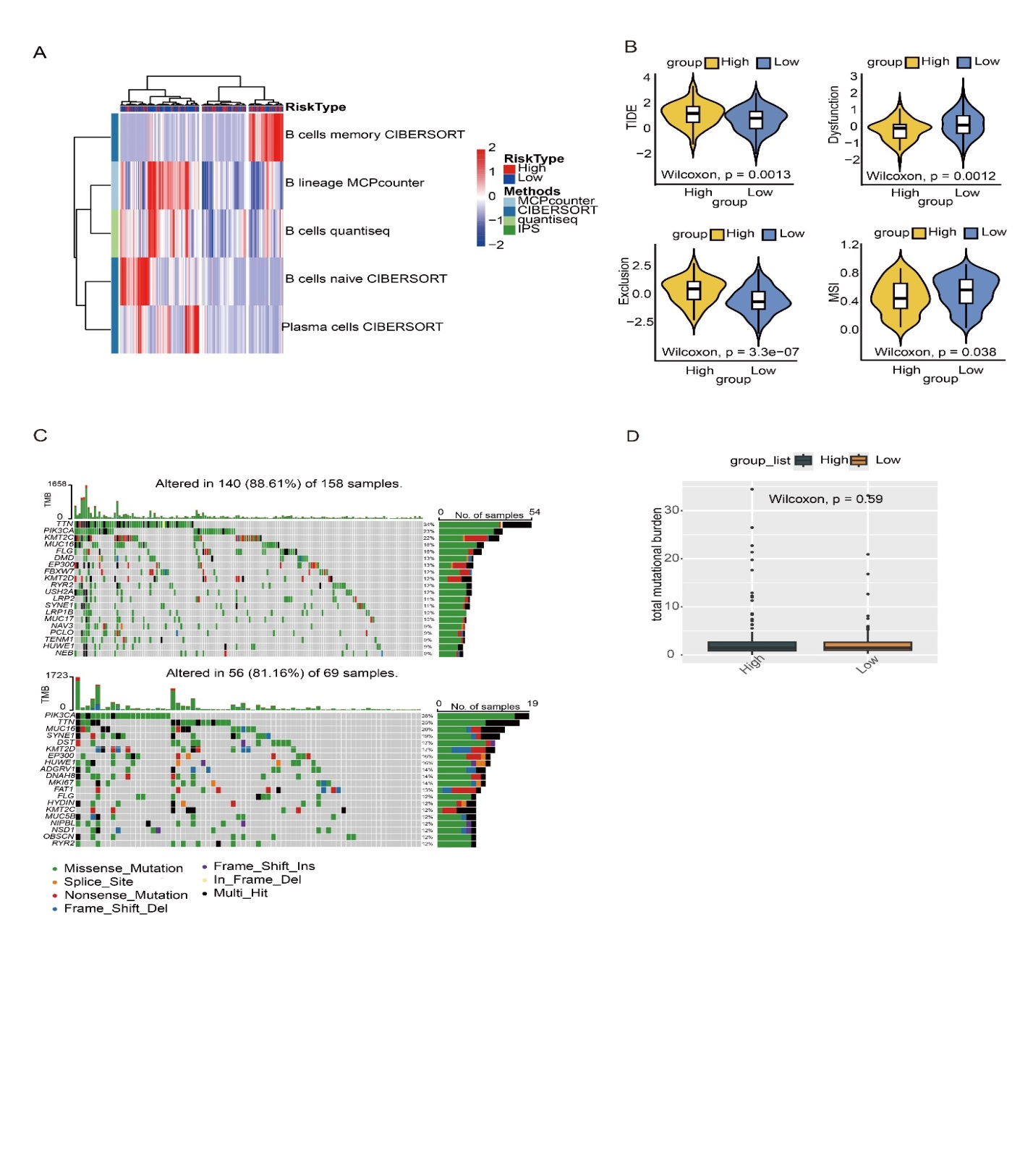


Figure S2

A The heatmap illustrates the clustering patterns of B cell infiltration between HRPS^high^ and HRPS^low^ groups, as determined through multiple analytical approaches.

B The violin chart displays the TIDE scores between the HRPS^high^ and HRPS^low^ groups.

C Waterfall charts visualize the somatic mutation maps of the HPRS^High^ and HPRS^Low^ groups.

D Box plots compared the total mutational burden between the two groups.
